# Cotton Seed in Intermittant Fever

**Published:** 1851-01

**Authors:** 


### ARTICLE IV.

*Cotton Seed, (Gossypium Herbaceum) as an Antiperiodic in Intermittent Fever.*

To the virtues and uses of this plant, already enumerated in the report on the "Indigenous Medicinal plants of South Carolina," by Dr. FRANCIS P. PORCHER, let me add its use in intermittent fevers.

The information is derived from Dr. W. K. DAVIS, of Monticello, Fairfield District, South Carolina, in reply to inquiries made by him as to the medicinal properties and uses of cotton seed tea in some of the forms of fever.

The use of cotton seed tea in fever originated with a planter in Newberry District, who has used it liberally among his negroes, and uniformly with success.

"I have never failed," he says. "to cure a patient, with a single dose of it, even where large doses of quinine have failed. Where the patient has been ill of third day fever and ague, and for months, in such cases success has followed its use."

Professor SHEPARD's analysis of cotton seed tea shows it to be composed of many inorganic matters, some of which may really possess great medicinal virtues in this disease.

The mode of using cotton seed tea is as follows: After having given a dose of calomel, the day or night previous to the attack, followed by castor oil in time to produce a cathartic effect before administering the tea, you put a pint of cotton seed with a quart of water, in a vessel, and boil it until half the water has evaporated. Put the patient in bed, an hour or two before the usual recurrence of the ague, and give him a gill of the warm tea to drink.

Without advancing any opinion in reference to its exhibition, whether for or against, I present it to the notice of the profession, as a remedial agent becoming popular in domestic use in the section of country mentioned, and, therefore, claiming investigation on the part of the medical profession.—*Charleston, S. C. Med. Jour.*

